# Topics of Public Interest

**Published:** 1916-07

**Authors:** 


### TOPICS OF PUBLIC INTEREST.

**Rank for Dental Surgeons.** Senator Pomerene has introduced an amendment to the army bill, providing that dental surgeons shall have the rank, pay, etc., of first lieutenant for the first five years of service, instead of 10, of captain for the next ten years instead of until 25 years, and thereafter, the rank of major. At present, dental surgeons serve under contract for three years and, thereafter, on passing an examination, are permanently commissioned as first lieutenant but ranking below all other first lieutenants. The survival of an old prejudice against dentistry, and indeed against medical specialism in general, is the only excuse for such failure of recognition of the value and dignity of professional service.

---

**Batavia Hospital.** 26 physicians of the Medical Society of the County of Genesee have petitioned the directors for the removal of the superintendent and assistant.

---

**Associate Members of the Cleveland Academy of Medicine.** These include 4 dentists, 7 pharmacists, 18 veterinarians, and 3 miscellaneous—medical teachers not having the M. D. degree. Will our western N. Y. academies follow this hint?

---

**Tuberculosis Hospital Not a Nuisance.** The City of Northfield, N. J., attempted to enjoin Atlantic Co. from erecting a tuberculosis hospital. The ruling of the N. J. Court of Chancery, 95 Atl. 745 was based on the lack of evidence that the hospital would be a source of danger and on the general principle that mere interference with market value of adjoining property was not sufficient.

---

**Ambulances for Syracuse.** Mr. Alexander T. Brown has presented an ambulance to the Hospital of the Good Shepherd and St. Joseph's and the Crouse-Irving Hospital have each bought one.

---

**Onondaga Co. Tuberculosis Sanitarium.** Considerable fault is found with the construction, in spite of the high cost of $600,000. $5,000 is to be spent for linoleum, on account of the poor condition of the floors. (Crouse-Irving Bulletin).

---

**Proportion of Able Bodied Men.** 40% of about 800 applicants for positions as firemen in Buffalo, have passed the physical tests. In several instances throughout the country, 50% has been found in candidates for the national guard. These figures are significant from the standpoint of preparedness.

---

**Warning.** We are advised that a very clever swindle is being worked by a young man calling on physicians in various sections of the country. He is fraudulently soliciting orders and collecting money for subscriptions to medical journals and for medical books published by various firms. He usually represents himself as a student, working his way through college and trying to get a number of votes to help him win a certain contest. He sometimes uses the names of L. D. Grant, H. E. Peters, R. A. Douglas and F. C. Schneider and he usually gives a receipt bearing the heading of some society or Association, such as United Students Aid Society; the Alumni Educational League; the American Association of Education, etc.

The description given of this swindler is—young man of the Jewish type, rather slender, with very dark hair combed straight back and shows his teeth plainly when talking.

The whole scheme is a fraud. The Societies mentioned do not exist. The idea is to collect money by offering special discounts and prices on medical books and journals and skip with the money.

This young man does not represent W. B. Saunders Company, whose name he frequently uses. He is a fraudulent subscription agent and physicians, generally, should be on the lookout for him.

---

**Health Insurance.** In response to public interest in health insurance the Massachusetts Legislature has created a commission to study social insurance with special reference to sickness. The state department of health and the bureau of statistics are directed to co-operate with the commission of nine members which will prepare a report and recommend the form of legislation to be introduced in January, 1917. California has a similar state commission already at work on this problem which is attracting wide attention since the introduction this year of bills for health insurance in Massachusetts, New York and New Jersey. Proponents of this legislation believe it will bring a movement for "health first" comparable to the safety first campaign which followed workmen's compensation for accidents.

---

**War and Meteorology.** Most scientific authorities deny the effect of explosives on rain fall, claiming in particular that it rains on the Fourth of July and on the days of battles with just about the same average frequency as on any other day, and that rain-making bombs have produced rain only by coincidence. We are quite disposed to admit that a few fire crackers or even a few hundred small arms can have no appreciable influence on precipitation. But it is not a priori absurd that the tremendous explosions of modern artillery, almost continuously for considerable periods and for distances of many miles, may have an appreciable action. In many instances, not only vibrations but air currents of considerable magnitude are brought to bear pretty directly on the lower cloud level. We trust that the matter will be studied from the standpoint of actual fact, not of credulity or incredulity. It should be remembered that statistics of average or aggregate precipitation do not entirely settle the question. The violence of precipitation at a given time, the accompaniment of electric disturbances, the frequency of rains by the week or month, are quite as important, indeed more important in their effect on human industry and enjoyment.

**Moss as a Substitute for Cotton.** The Muench. Med. Woch. states that, owing to the shortage of cotton—which is needed for explosives more acutely than for alleviating the results of explosives—sphagnum (peat moss or turf) which is abundant in German moors, is largely used as a dressing. This absorbs 10 times its weight of water, cotton only 6 times. It will stand half an hour's sterilization by heat without damage. It may be directly applied and has the advantage over cotton that it does not stick in dry discharges as does cotton. It may also be used inclosed in gauze, as packing. Apparently it is to some degree directly haemostatic, as well as a better absorbent than cotton. The plant, while originally growing from a stalk seems to absorb its nourishment mainly through the leaves, the stalk soon dying. The cells of the leaves are approximately, alternately chlorophyl-containing and chlorophyl-free, the latter cells being larger and capable of absorbing either water or air as required in the growth of the plant while, in the surgical use of the moss, these chlorophyl-free cells, determine its remarkable absorptive capacity.

**The Picnic of the Buffalo Health Dept.** was held at Crystal Beach, June 17.

**Harrison Law Ruling.** The U. S. Supreme Court, Justices Hughes and Pitney dissenting, has ruled that the provision making it unlawful for anyone not registered to have opium in his possession applies only to dealers and not to users. This ruling, of course, renders the law ineffective to a large degree but is based on the principle that "it would not do to strain the powers of the U. S. almost if not quite to the breaking point." It will be remembered that, at the outset, we pointed out the ultimate danger of legislation on matters of morals and ethics and the impropriety of exceeding the constitutional powers of the central government by the quibble of introducing a scheme of taxation, itself troublesome and unwarranted. Already, in Buffalo, Detroit and probably other cities, the local authorities have been handicapped in prosecuting cases by this new ruling.

---

**Radium Cure of Cancer.** A case of facial cancer in a man 102 years old is reported from Kane, Pa., one eye being lost.

---

**The Batavia Woman's Hospital Assn.** has voted to install a new heating system, with a separate boiler building.

---

**Columbus Hospital of Buffalo** is being enlarged by a brick addition 2 stories high, with basement, increasing the capacity by 30 beds, at a cost of $25,000. $10,000 is also being expended on other improvements, including X-ray laboratory, laundry, enlargement of operating room, etc.

---

**Immigration Statistics.** For the year ending June 30, 1915, the net increase of immigration over emigration was 50,070, the smallest for a great many years. For the first 10 months of the fiscal year 1915-16, the immigration was 291,527 and the emigration 212,478, a net increase of 79,049. Apparently the breathing spell due to the War, will not last long.

---

**Health Insurance.** The U. S. Public Health Service, in advocating a governmental system, states that 2.5% of employes in American industries are constantly incapacitated and that the average worker loses 9 days a year from sickness.

---

**The N. Y. State Dept. of Health** has received an appropriation of $65,000 for the erection of a new laboratory to take the place of the present quarters in an old stable that has been condemned as unsanitary and that has not afforded even sufficient room for convenient arrangement of time.

---

**The J. N. Adam Memorial Hospital** at Perrysburg, will be increased by 30 beds, a temporary shack for winter and more tents for summer. The directors and the councilmen of Buffalo inspected the hospital June 10.

---

**Warning.** The State Commissioner of Health has issued a letter to the effect that facilities are now acquired for enforcing the law regarding the reporting of contagious diseases, births, etc. It should be remembered that practically all infectious diseases must be reported, including trachoma, tuberculosis, puerperal septicaemia; that previous attendance by another physician or the fact that one is a consultant does not necessarily relieve from responsibility. It is a safe rule to telephone the local authorities in the case of any unusual condition found and to ask one's self whether the circumstances are such that the health officials would be interested.

---

**The Open Air Camp for Tuberculous Patients** has been opened for the season, under the auspices of the Buffalo Association for the Relief and Control of Tuberculosis. Applications should be made at the Dispensary, 175 E. Swan St., Seneca 1270-W.

---

**Anthrax.** A case was reported to the Buffalo Health Dept. in May, in the person of a street car conductor, supposed to have been infected from a glove. Recovery ensued.

---

**Infant Mortality.** The N. Y. Milk Committee (105 E. 22, N. Y.) have prepared a table showing the mortality of infants under one year for 144 cities of the U. S. The averages of 1906-10 for about a third of these cities, indicate that the supposedly "normal" mortality of infants was about 125 per 1000 born. The lowest mortality for a large city was 91 for Minneapolis; Baltimore had a mortality of 228 and Troy, the enormous one of 267. The only small mortality rate reported was from Brookline: 59.3: 1000. Here, of course, the total population was: 27,792, the births and infantile deaths very low in actual numbers, since this is probably the richest incorporated place in the world, proportionately to population, and the one that has the smallest number of families of small means. In fact, about the only poor persons in it are domestic servants. While there have been and will be considerable fluctuations in infantile mortality since 1910, and variations not entirely explained by kind of population, climate, etc., it is noteworthy that for 1915, there was not a single place that reached the 200:1000 limit; only 8 that passed 150 and all of these comparatively small places, Nashville (population 110,000) being the largest, while all of the 8 had unfavorable conditions, such as a large industrial population in mills, etc., or excess of colored population. 23 of 50 cities above 100,000 in population, exceeded 100:1000 infantile mortality and, of the 144 places listed, only 50 exceeded this rate. An approximate average by places, shows that 75:1000 is a rate already attained. Seattle (237,000 inhabitants) had a rate of 53.1 and Omaha (124,000) a rate of 47.1, these being less than half of the average rates for the years 1906-10. Hence the commission issues the slogan that

"NO COMMUNITY WITH AN INFANT MORTALITY OF OVER 50 PER 1000 BIRTHS CAN CLAIM THAT ITS BABIES ARE GETTING A SQUARE DEAL."

---

**Brooks Memorial Hospital** graduated 10 nurses, May 24. Dr. Thew Wright of Buffalo delivered an address.

---

**Adenoids at Perry.** Dr. L. W. Jones of Rochester was engaged to operate on school children, May 29, rooms being fitted up for operating and hospital purposes.

---

**Telephone Rates in Detroit.** A. Business: 150 calls a month, $4.50, extra messages 2 cents; party lines, 50 calls, $2.50; unlimited service, single line, $6; unlimited service, 2-party line, $5. B. Residence: single line, $3.50; 2-party line, $3; 4-party line, $2. The Bell Co. has no competitor and is attempting to raise rates.

---

**Thymol From Horse Mint.** Fresh, entire herbs, Putnam and Volusia Cos., Fla. 1907, yielded 1-2 parts per 100 of essential oil, the oil consisting of 56-62% of phenols which were almost entirely thymol. Wild plants consist mainly of stems and when dry, yield practically no oil. In 1915, by cultivation, the yield of oil had been increased to about 4:1000 and the oil yielded 74% of phenols. Directions are given for cultivation, distillation, etc., and the estimate is made that $16 per acre may be obtained. S. C. Hood, Bull. 372, U. S. Dept. of Agriculture.

---

**Army Medical Corps Examinations** will be held at points to be announced July 17 and Aug. 14. Over 100 vacancies are to be filled. Citizens, 22-30 years old, with a medical degree and having had a year's interne service are eligible. Apply to the Surgeon General, U. S. Army, Washington, D. C.

---

**Office Building for Buffalo Physicians.** It is proposed to erect a real medical office building for Buffalo, on a scale with similar buildings in Denver, Chicago and other cities. Provision will be made for housing the Academy of Medicine or such other society as may be inclined to accept the offer and to provide a kitchen, banquet hall, etc., for social meetings. Parking space for automobiles, abundance of light that cannot be interfered with by any subsequent building and accessibility will be considered in selecting a site. We understand that capital will be raised outside the profession, as a business investment, to secure a return from rentals, though it is probable that physicians may also invest if they so desire. We would be pleased to hear from our readers as to how they regard this proposition.

---

**Saratoga Development.** Gov. Whitman, in his address to the State Society, estimated that 25,000 or more Americans have annually visited European spas, spending ten millions a year, beside passage money. The Reservation has been placed under the control of the Conservation Commission and the State will appropriate funds for the development of Saratoga as the profession by advising patients to seek relief in their own country, justifies such expense.

---

**The Preparedness Parade** for Buffalo, June 24, included 48,100 men and women, exclusive of bands and guardsmen. 2600 men paraded with military organizations and 1000 as bands, aids, etc., making a total of 51,700, well above 10% estimate of militia in the total population. 320 physicians marched, notwithstanding the fact that a considerable number were already on duty with the national guard or out of town. Unfortunately, 109 cases of drunkenness ascribed to patriotism were disposed of in the courts the next day. One point should be clearly kept in mind, especially by physicians, for their own guidance and that of others whom they may be called to advise. At present, a comparatively small number of men are needed and it is scarcely likely that a war with Mexico will require more than a million men—1% of the total population or 10% of the available military strength. This number can easily be raised without withdrawing from civil life those who are required for the maintenance of the various industrial and other activities of the community or those whose absence and, still more, whose death or permanent disability will cost the country more than their value as military units. True patriotism should take these facts into account and should be based on a wise consideration of actual personal conditions, rather than on emotional sacrifice. We trust, however, that as a general preparedness for more remote danger, every unit of the National Guard, mobilized for service, may be replaced by some sort of a Home Guard, of full strength and promptly drilled to the fullest efficiency possible under peace conditions for the National Guard.

---

**The International Joint Commission** to consider the pollution of boundary waters between the U. S. and Canada, met in Buffalo in June. Buffalo will present, within six months, a plan for sewage disposal and also for water filtration. It is estimated that about 3 million dollars will be required to carry out an ideal plan. This will be a considerable strain upon the finances of the city, corresponding to about a quarter of the total expense of city government for a year.

---

**Dental Preparedness.** A national league of dentists has been instituted by Drs. J. Wright Beach, Herbert A. Pullen and M. Button Eschelman of Buffalo, the enrollment being already over 1000. The objects are to give free dental services to recruits, so as to bring them into perfect physical condition so far as the teeth are concerned, and to form local dental units for the treatment of wounds of the jaws which have proved far more common in the present war than in any other.

---

**Automobile Registration.** Up to June 10, the N. Y. state licenses number 247,397, an increase of 59,972 over the period of 1915. 16,698 motorcycle licenses have been issued.

---

**Anti-Typhoid Vaccination** will be applied to all N. Y. State National Guard soldiers. Enough for 4000 persons was issued by the State Health Dept. June 21, many have already been immunized and as the Dept. has foreseen the need of sufficient vaccine, this element of preparedness will have been carried out completely. The Guard of other states has been vaccinated to a large degree.

